# Topological Characteristics Associated with Intraoperative Stimulation Related Epilepsy of Glioma Patients: A DTI Network Study

**DOI:** 10.3390/brainsci12010060

**Published:** 2021-12-31

**Authors:** Jianing Yang, Chunyao Zhou, Yuchao Liang, Yinyan Wang, Lei Wang

**Affiliations:** 1 Department of Neurosurgery, Beijing Tiantan Hospital, Capital Medical University, Beijing 100071, China; yjn1997666@163.com (J.Y.); 112019000326@ccmu.edu.cn (C.Z.); yuchao_liang@bjtth.org (Y.L.); 2 Beijing Neurosurgical Institute, Capital Medical University, Beijing 100071, China

**Keywords:** intraoperative stimulation-related epilepsy, awake craniotomy, glioma, diffusion tensor imaging, graph theoretical analysis

## Abstract

*Background*: Awake craniotomy with intraoperative stimulation has been utilized in glioma surgical resection to preserve the quality of life. Epilepsy may occur in 5–20% of cases, leading to severe consequences. This study aimed to discuss the mechanism of intraoperative stimulation-related epilepsy (ISE) using DTI-based graph theoretical analysis. *Methods*: Twenty patients with motor-area glioma were enrolled and divided into two groups (Ep and nEp) according to the presence of ISE. Additionally, a group of 10 healthy participants matched by age, sex, and years of education was also included. All participants underwent T1, T2, and DTI examinations. Graph theoretical analysis was applied to reveal the topological characteristics of white matter networks. *Results*: Three connections were found to be significantly lower in at least one weighting in the Ep group. These connections were between A1/2/3truL and A4ulL, A1/2/3truR and A4tR, and A6mL and A6mR. Global efficiency was significantly decreased, while the shortest path length increased in the Ep group in at least one weighting. Ten nodes exhibited significant differences in nodal efficiency and degree centrality analyses. The nodes A6mL and A6mR showed a marked decrease in total four weightings in the Ep group. *Conclusions*: The hub nodes A6mL and A6mR are disconnected in patients with ISE, causing subsequent lower efficiency of global and regional networks. These findings provide a basis for presurgical assessment of ISE, for which caution should be taken when it involves hub nodes during intraoperative electrical stimulation.

## 1. Introduction

Glioma is the most common neoplasm of the central nervous system, and its standard treatment includes surgical resection and subsequent chemo-radiotherapy [1,2]. Awake craniotomy with intraoperative electrical stimulation has been used to identify the sensorimotor cortex and other functional regions with high efficiency and accuracy, which is considered important to preserve the quality of life to the utmost [3,4,5]. However, 5–20% of cases may experience seizures during this procedure, interfering with patient compliance and causing poor outcomes [6,7]. Although treatments such as propofol sedation and cold Ringer’s lactate may reduce the seizure onset, the occurrence of intraoperative stimulation-related epilepsy (ISE) can lead to serious postoperative complications, such as motor deficits or cognitive impairment [6,8]. It is currently acknowledged that preoperative epilepsy, tumor location, repeated stimulation, and high stimulation current are predisposing factors for ISE [9]. However, the mechanisms of ISEs are rarely studied. ISE seems random and unpredictable; hence, it is one of the main risks of AC. Therefore, the prediction and prevention of ISE need to be investigated.

The mechanism of epilepsy, though generally researched, remains unclear. Brain networks are complicated in terms of anatomical substance and physiological functions [10], and most subtypes of epilepsy work in a network pattern [11,12,13]. Most general seizures are related to sensorimotor cortex discharges and impair functional or structural connections in several networks. Therefore, graph theory, which focuses on topological patterns, can be utilized to analyze the structural alterations in the brain networks of epilepsy patients. However, the association between ISE and alterations in subcortical networks and their topological attributes remains unknown.

DTI is an advanced technique to measure subcortical structures non-invasively quantitatively [14,15], which can be used to reveal white matter structure changes under the conditions of multiple brain functional or structural disorders. Abnormalities in DTI networks can represent the dysfunction of brain networks in patients with epilepsy. Therefore, diffusion metrics and graph-theoretical attributes could provide details on the characteristics of epilepsy [10,16,17], normally reflected as global efficiency decreased, and the shortest path length increased in patients with epilepsy [18,19]. Furthermore, some topological attributes, including small-world, have a synchronic effect in epilepsy development by stronger clustering co-efficiency and shorter path length [20]. Therefore, identifying the characteristics of subcortical networks in patients with ISE will help understand the mechanism of seizure onset better and increase the prognosis of suspectable patients.

Using the DTI technique and graph theory, the main objective of this study is to identify ISE related characteristics in the perspective of network.

## 2. Materials and Methods

The study was conducted according to the guidelines of the Declaration of Helsinki, and approved by the Institutional Review Board of Beijing Tiantan Hospital. Written informed consents have been obtained from the patients involved in the study to publish this paper.

### 2.1. Participants

A total of 20 patients diagnosed as left-sided frontal gliomas from 2018 to 2020 at our hospital were retrospectively recruited. All patients were included according to the following criteria: (a) older than 18 years, (b) pathologically diagnosed as WHO II-IV glioma according to the WHO (2016) standard for CNS neoplasm, (c) more than 9 years of school education, and (d) no history of presurgical biopsy, radiotherapy, or chemotherapy. The exclusion criteria were as follows: (a) contraindications for MRI, (b) head motion > 2 mm in translation or 3° in rotation, and (c) history of general seizure onset or AED usage before MRI acquisition. Finally, 20 patients (nine men) were enrolled in the study. Additionally, a group of 10 healthy participants matched by age, sex, and years of education was also included.

### 2.2. MRI Acquisition

All MRI data were acquired using a MAGNETOM Prisma 3T MR scanner (Siemens, Erlangen, Germany). Anatomic images were collected with T1 magnetization-prepared rapid acquisition of gradient echo (TR = 2300 ms, TE = 2.3 ms, flip angle = 8°, the field of view, FOV = 240 × 240 mm, voxel size = 1.0 × 1.0 × 1.0 mm^3^, slice number = 192). T2-FLAIR sequences were used to acquire the tumor mask (TR, 3200 ms; TE, 87 ms; FA, 150°; FOV, 220 × 220 mm; voxel size = 0.9 mm × 0.9 mm × 5 mm; slice number = 25). DTI data were acquired using single-shot, echo-planar imaging sequence (TR = 6000 ms, TE = 103 ms, axial slices = 75, resolution = 2.0 × 2.0 × 2.0 mm, flip angle = 75°; FOV = 230 mm × 230 mm; voxel size = 2.0 mm × 2.0 mm × 2.0 mm, number of directions = 30, b = 0/1000 s/mm^2^, EPI factor = 154). All MRI data were acquired within 48 h before surgery.

### 2.3. DTI Data Preprocessing

Preprocessing and analysis of diffusion metrics were performed using the PANDA toolbox (https://www.nitrc.org/projects/panda/ (accessed on 18 June 2018)). The detailed preprocessing, tractography, and network construction steps were clarified by Cui et al. [21]. Briefly, after converting raw DICOM data to a Nifti format, PANDA implemented steps to extract basic DTI metrics, including brain mask extraction, eddy current effect correction, averaging multiple acquisitions, diffusion tensor calculation, and metrics production. Atlas-based FACT deterministic fiber tracking was implemented with the pre-set motor-sensory network (angle threshold = 45°, FA threshold = 0.2).

### 2.4. Operation and Stimulation Protocol

All enrolled patients had undergone awake craniotomy. All surgery were performed by two chief neurosurgeons with similar seniority in our department. The surgical approach varied according to tumor location. The Brainlab navigation system (Brain Lab, Munich, Germany) was utilized in every surgery. The anesthesia procedure were implemented by experienced anesthesiologists. Anesthesia begun with routine monitoring and followed by anesthesia induction and mechanical ventilation. For anesthesia induction, we used propofol, sufentanil and remifentanil while propofol and remifentanil were used in maintainence. After the completion of craniotomy, the anesthesiologist would temporarily stop the usage of propofol and remifentanil. Stimulation procedure was performed when the bispectral index (BIS) was over 80. The motor-sensory cortex was identified with an Ojemann stimulation system with a 5 mm diameter (intensity, 1–6 mA; frequency, 60 Hz; square wave). The spatial location of the stimulation points of each participant is relatively fixed. The stimulation current began at 1 mA and was increased by 0.5 mA until the patient showed unconscious limbal movements (precentral gyrus) or transient numbness (postcentral gyrus), thus establishing the stimulation threshold. Once the stimulation threshold was established, the stimulation current was unchanged and used to identify the motor cortex and subcortical structures. The stimulus duration for identification was 1 s for motor- and sensory-related cortices. No site was stimulated continuously. Iced Ringer’s solution was used for ISE occurrence. If epilepsy lasted over 10 s, benzodiazepine was administered, and the operation had to be temporarily discontinued. ECoG was not utilized in this process.

### 2.5. Tumor Region of Interest Extraction

**Figure 1:**
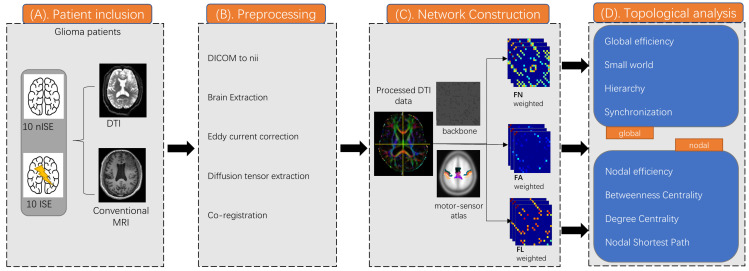
Methods and protocol. (**A**)Twenty patients (10 with ISE and 10 without ISE) were included and experienced DTI and conventional MRI. (**B**) Diffusion metrics was preprocessed by PANDA toolbox. (**C**) Construct a white matter connectome matrix weighted by FN, FA, and FL. (**D**) Global and nodal topological properties were calculated and analysed.

**Figure 2:**
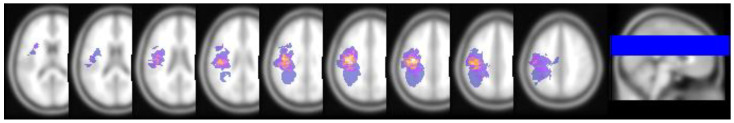
Normalized tumor overlap map. Overlapping map of all normalized tumor ROI. Voxel color indicates the number of overlapping cases, from 1 (blue-purple) to 12 (light yellow).

The glioma regions were manually segmented independently by two experienced neurosurgeons. For low-grade gliomas, ROIs were extracted based on T2-FLAIR, while for anaplastic glioma and glioblastoma, ROIs were extracted based on T1-contrast enhancement. Peritumoral edema was carefully avoided in all cases. If the masked tumor region varied over 5% between the two surgeons, a neuro-radiologist with 20-year-experience would make the final decision. All tumor masks were then normalized to the MNI standard space using the clinical toolbox package in SPM8 (http://www.fil.ion.ucl.ac.uk/spm/software/spm8/ (accessed on 18 June 2018)) (Figure 1B). Finally, all normalized tumor masks were overlapped to create a tumor volume overlapping image (Figure 2).

### 2.6. Network Construction

The nodes considering the motor-sensory network were extracted from an open access brain atlas, “brainnetome atlas” (http://www.brainnetome.org/ (accessed on 5 November 2019)). To avoid neurovascular uncoupling or a tumor-occupying effect, regions invaded by gliomas were excluded. To construct a white matter (WM) connectome matrix, deterministic fiber tracking was used to track the WM connections for all possible pairs of nodes. Consequently, for each subject, a 22 × 22 white matter connectome matrix was constructed, and each edge in the matrix was weighted by the averaged fiber number (FN), fractional anisotropy (FA), and matrix length (FL).

### 2.7. Graph Theoretical Measures

Before each matrix was delivered into the theoretical graph analysis, a backbone method with a threshold above 75% was applied to minimize error connections caused by normalization error or partial volume effect. Global and nodal topological properties, including global efficiency, local efficiency, nodal efficiency, degree centrality, and small-world properties (gamma, lambda, and sigma), were calculated using the GRETNA toolbox (https://www.nitrc.org/projects/gretna/ (accessed on 5 November 2019)) [22] for each patient and healthy control (The algorithm in detail was clarified in Supplementary Material). Briefly, global efficiency signifies the ability of information conduction, and the globally shortest path length represents the minimum number of passing edges between every pair of nodes. Usually, a higher global path length is always accompanied by a lower global efficiency. Nodal properties can characterize the local efficiency of information conduction. Nodes with a higher degree of centrality usually play a more critical role in the network. Detailed formulas of the topological properties are shown in Supplementary Materials. Binary, FA, FN, and FL weighted networks were used to calculate each topological property.

### 2.8. Statistical Analysis

Clinical characteristics were compared between the ISE group, non-ISE group, and controls using one-way ANOVA and Fisher’s exact test in R (https://www.r-project.org/ (accessed on 5 November 2016)). All network-related statistics (FA, MD, matrix length and global and nodal properties) were regarded as non-parametric statistics. Thus, for inter-group comparison on edges of netwrok, we used NBS toolbox (https://www.nitrc.org/projects/nbs/ (accessed on 18 June 2020)) to carry out non-parametric ANOVA based on network-based permutation method (*n* = 5000). As for comparisons of topological properties, z-transformation was performed on all global and nodal properties. Further analysis was based on the z-scored statistics to ensure the parametric intergroup comparisons. One-way ANOVA with false discovery rate correction and the post-hoc test was used to calculate the differences in inter-group WM connections and topological properties.

## 3. Results

### 3.1. Demographic Characteristics

A total of 20 patients (nine men) with frontal-lobe glioma and 10 healthy controls (five men) were enrolled in this study. Patients were divided into two groups according to whether epilepsy onset was induced by intraoperative stimulation. The three groups were as follows: glioma patients with intraoperative stimulation-induced epilepsy (Ep, *n* = 10), glioma patients without intraoperative stimulation-induced epilepsy (nEp, *n* = 10), and control group (con, *n* = 10) (Table 1). The age of patients ranged from 19 to 64. There were 11 males and nine females. All patients and controls had 12–18 years of education. The time from initial radiological diagnosis to surgical treatment was between 10 to 60 days except two patients (one in EP and one in nEp) with over 240 days of clinical observation. A total of six patients presented presurgical language deficits (three Ep, three nEp) including anomia and delayed speech. Six patients presented motor deficits (3 Ep, 3 nEP) that manifested as limbal weakness. No intergroup significant differences were observed in age, sex, education, diagnosis time, histology, IDH status, tumor volume, preoperational KPS and stimulation current (*p* > 0.05). All tumor ROIs showed no obvious discrepancy between the two neurosurgeons.

### 3.2. Connections Differences

**Figure 3:**
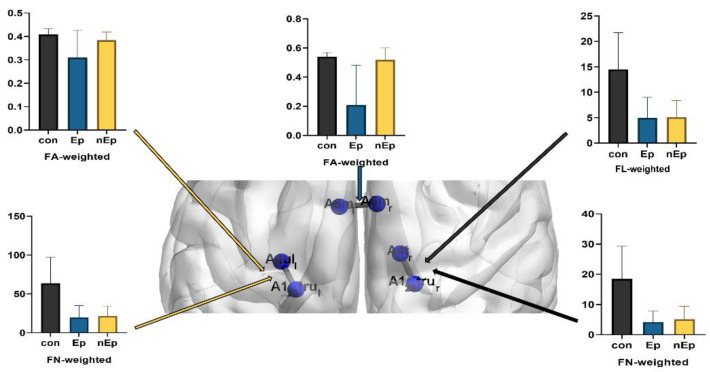
Connection differences.

Except for six nodes that were invaded by the tumor, all the connections in motor-sensory networks were compared among the Ep, nEp, and control groups with binary and weighted (FA, FL, and FN) networks. Among them, three connections showed significant intergroup differences. These are the connections between the left medial area 6 (A6mL) and right medial area 6 (A6mR) in the bilateral superior frontal gyrus, left area 4 (A4ulL) representing the left upper limb region in the right precentral gyrus and left area 1/2/3 (A1/2/3truL) representing the left trunk region in the right postcentral gyrus, and right area 4 (A4tR) representing the right trunk region in the left precentral gyrus and right area 1/2/3 (A1/2/3truR) representing the right trunk region in the left postcentral gyrus, respectively. (Table 2, Figure 3) Relative to the control group, Ep group showed lower FA and FN (FA, *p* = 0.017; FN, *p* = 0.0014) in the A1/2/3truL and A4ulL connection. In addition, the FN was also significantly lower in the nEp group than in the control group (*p* = 0.0017). As for the connection between A1/2/3truR and A4tR, FN in the control group was significantly higher than in both the Ep and nEp groups (*p* = 0.0008, *p* = 0.0018, respectively). FL also decreased in the Ep and nEp groups compared to the control group (*p* = 0.0017 and *p* = 0.0014, respectively). Another connection was found between A6mL and A6mR. The Ep group exhibited lower FA than both the control and nEp groups (*p* = 0.0012 and *p* = 0.0028, respectively), but no significant differences in FA between the nEp group and control.

### 3.3. Global Properties Differences

**Figure 4:**
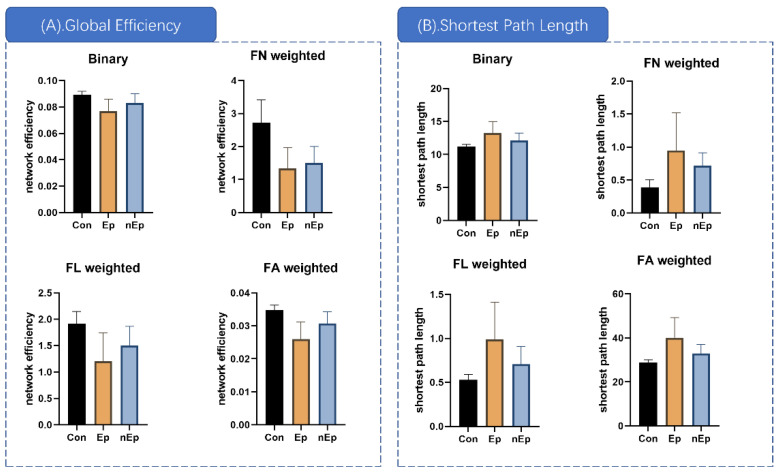
Global properties differences. (**A**) The global efficiency of Ep group markedly decreased than control in binary, FA, FN, and FL. The global efficiency og nEp group is also significant decreased than control in the above four weightings. Ep group only showed lower global efficiency in FA weighting comparing to nEp. (**B**) The shortest path lengths of Ep group showed noticeable increase than the control group in the above four weightings. Likewise, the shortest path length of nEp group is also significantly higher than control. Ep group only showed increase of shortest path length in FA weighting comparing to the nEp.

Global efficiency, small-world properties, hierarchy, synchronization, and rich clubs were selected to measure inter-group global differences using one-way ANOVA. (Table 3) Global efficiency and shortest path length showed significant differences in binary and all three weighted networks (*p* < 0.01, *p* < 0.01, respectively). Significant global attributes were further analyzed using post-hoc analysis. The global efficiency in the Ep group was markedly decreased compared to that in the control group in binary, FA, FN, and FL (*p* < 0.01 in binary, FA, and FN and *p* = 0.0012 in FL, Table 3, Figure 4A). The difference between the control and nEp groups was also significant in the above four weightings (*p* = 0.153 in binary, *p* = 0.0041 in FA, *p* < 0.001 in FN, and *p* = 0.0072 in FL, Figure 4A). Compared to the nEp group, the Ep group showed lower global efficiency in FA weighting (*p* = 0.031, Figure 4A). Considering the shortest path lengths, the Ep group showed a noticeable increase in the shortest path length compared to the control group in the above four weightings (post-hoc, *p* = 0.0032 in binary, *p* = 0.0011 in FA, *p* = 0.0070 in FN, and *p* = 0.0032 in FL, Table 3, Figure 4B). Likewise, the shortest path length of the nEp group was also significantly higher than that of the control (post-hoc, *p* < 0.0203 in binary, *p* = 0.0055 in FA, *p* < 0.001 in FN, and *p* = 0.0160 in FL, Figure 4B). Compared to the nEp group, the Ep group showed a marked increase in the shortest path length in FA weighting (post-hoc, *p* = 0.0363, Figure 4B).

### 3.4. Nodal Properties Differences

**Figure 5:**
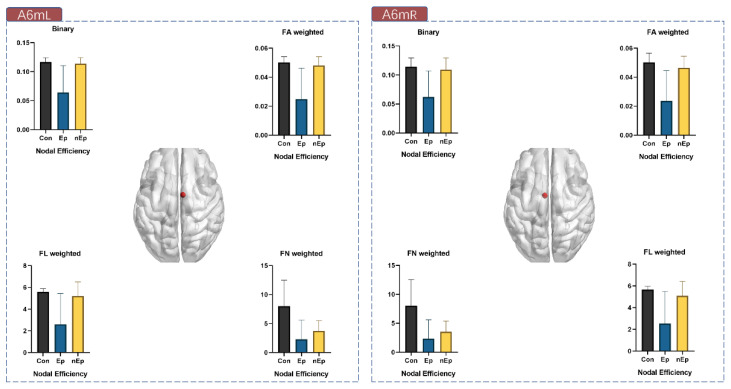
Nodal efficiency of A6mL and A6mR in all four weightings.

**Figure 6:**
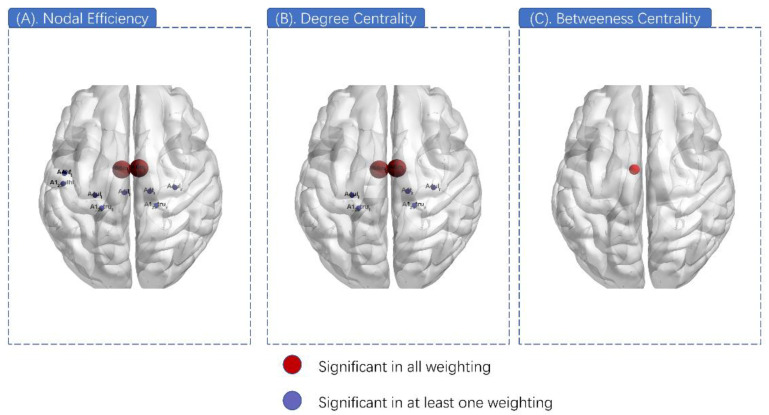
Nodal properties differences. (**A**) Ten nodes exhibited a significant difference in at least one weighting of nodal efficiency (pueple). A6mL and A6mR exhibited a significant difference in all four weightings of nodal efficiency (red). (**B**) Seven nodes exhibited a significant difference in at least one weighting of degree centrality (purple). A6mL and A6mR exhibited a significant difference in all four weightings of degree centrality (red). (**C**) A6mL also exhibited inter-group disparity in betweenness centrality.

The nodal properties that presented local efficiency related to the motor-sensory network were measured, including nodal efficiency, betweenness centrality, degree centrality, nodal shortest path length, and clustering coefficient. Among them, nodal efficiency and degree centrality were found to have significant inter-group differences. In nodal efficiency, 10 nodes exhibited significant differences (A6mL, A6mR, A4tR, A4ulL, A4ulR, A4ullL, A123truL, A123truR, A123ulhfL, and A4hfL) in at least one weighting (Table 4). Among them, A6mL and A6mR showed marked differences in all four weightings (Figure 5). As for degree centrality, the analytic results seemed similar to the nodal efficiency, with seven nodes illustrating significant differences (A6mL, A6mR, A4tR, A4ulL, A4ulR, A123truL, and A123truR) and A6mL and A6mR also showed a markable difference in total four weightings from among (Table 5). In addition, A6mL also exhibited intergroup disparity (*p* = 0.0003) in betweenness centrality (Figure 6).

**Figure 7:**
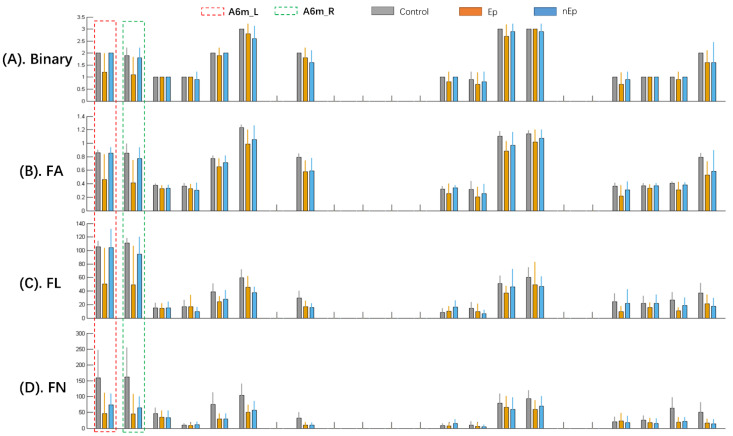
The degree centrality (DC) of the nodes in the motor-sensory network. A6mL and A6mR showed relatively higher DC than the other nodes in both healthy controls and non-ISEs. In addition, the Ep group showed a marked decrease of DC in the A6mL and A6mR. Each three-color bars represents the DC value in one single node. (**A**) Nodal DC values of binary networks. (**B**) Dc values of FA weighted networks. (**C**) DC values of FL weighted networks. (**D**) DC values of FN weighted networks.

We further analyzed the nodal efficiency (Table 6) and degree centrality (Table 7) of A6mL and A6mR by subsequent post-hoc tests in four weightings. Compared to the control group, the Ep group showed markable decrease of nodal efficiency in all four weightings of both A6mL and A6mR (*p* = 0.003, 0.002 in binary, *p* = 0.002, 0.001 in FA, *p* = 0.004, 0.004 in FN, and *p* = 0.004, 0.003 in FL, respectively). Significant differences were also observed between the Ep and nEp groups in binary, FA, and FL weightings of both A6mL and A6mR (*p* = 0.004, 0.007 in binary, *p* = 0.004, 0.005 in FA, and *p* = 0.0016 and 0.023 in FL, respectively). As for the comparison between nEp and control, only FN showed a decrease in both A6mL and A6mR (*p* = 0.011 and 0.009, respectively). The results of degree centrality analyzed by post-hoc test among the three groups were very close to the nodal efficiency mentioned above, showing the significant difference between Ep and control of both Am6L and A6mR (*p* = 0.004, 0.006 in binary, *p* = 0.003, 0.001 in FA, *p* = 0.004, 0.004 in FN, and *p* = 0.004, 0.003 in FL, respectively). Significant differences of degree centrality were also observed between the Ep and nEp groups in binary, FA, and FL weightings of both A6mL and A6mR (*p* = 0.004, 0.017 in binary, *p* = 0.004, 0.007 in FA, and *p* = 0.0011 and 0.034 in FL, respectively). Furthermore, when comparing the degree centrality among nodes, A6mL and A6mR had the highest degree centrality value in FN and FL networks and a relatively high degree centrality value in binary and FA networks (Figure 7).

## 4. Discussion

DTI-based network analysis can be informative for evaluation of subcortical conditions of the whole brain. Since preoperative DTI combined with neuronavigation has been widely used in surgical planning, the WM network analysis method shows generalisability. In the current retrospective study, all 20 subjects were divided into two groups (Ep and nEp) according to ISE occurrence. The comparison of connections in motor-sensory networks among the three groups showed partial derangement in ISE patients. The following analysis of global topological properties exhibited remarkable alterations in ISE patients, with worse network efficiency and longer shortest path length. Considering the robust results on global topology, nodal properties were further measured, among which A6mL and A6mR exhibited a marked decrease in nodal efficiency and degree centrality in the Ep group.

The current study involved patients with left-sided motor area glioma, with or without ISE. We only considered 28 subregions of the motor-sensory network (brainnetome 246 atlas). ISE always occurs around the stimulation point, normally located at or near the motor cortex (or SMA), and the motor-sensory network is more vulnerable to major epileptic seizures. Further, controlling the tumor-related mass effect is a major challenge in the network analysis of glioma patients. The tumor’s oppression, invasion, or infiltration alters its surrounding anatomical structure, causing inaccuracy in the normalization procedure. As a result, it will cause mistakes in regions of interest in locating and atlas-based fiber tracking. Therefore, we created a tumor-overlapping map, and all subregions within the map were avoided. Thus, 22 subregions were included in the analysis.

Our results demonstrated several subcortical connections that showed a tendency for degeneration in FA, FN, and FL. Two connections (A6mL to A6mR and A4ulL to A123truL) showed decreased FA in the Ep group compared to nEp and healthy controls. FA is considered the most sensitive parameter for measuring white matter connectome abnormalities in SMA-originated epilepsy, as decreased FA equaled decreased orientation and fiber density [23]. In the current study, the main difference between the Ep group and the other two groups is the FA-weighted connections, especially between A6mL and A6mR. The connection between interhemispheric SMA has been proven to be reduced in glioma patients [24], indicating that this connection is vulnerable in the procedure of tumor occurrence, and the impairment of the white matter connection might be related to the occurrence of epilepsy [23].

Brain network dysfunction is associated with the onset of epilepsy [12,25,26]. In the current study, all individual networks exhibited small-world characteristics, indicating that although they were partially involved in tumor or tumor-related edema, the entire network of these patients still exerts better efficiency with long-distance connections [20,27]. The rise of the globally shortest path length is always associated with a decrease in global efficiency, indicating disruption of the brain network in ISE patients [28,29]. The network damage is proven to be continent and dynamic, further increasing the network disruption and leading to a vicious spiral [12,30]. Together with the very topological features of several decreased connections, the above results demonstrate both local segregation and global disruption in ISE patients, which is in line with prior network studies [19]. The white-matter connections mentioned above may be one of the causes of collapsed networks [19]. Eddin et al. [31] also assessed the network efficiency of pediatric epilepsy and found it to be significantly lower. The authors analyzed the partial functional network of these children, discovering that patients tended to activate the whole brain network. At the same time, the healthy controls only made use of a smaller independent network to achieve the same task [31]. Hence, the less efficient network may perform worse in information transferring from there needs to trigger a larger subnetwork, suggesting less precision and more randomness. When white matter degeneration or reorganization occurs, the consequent reduction in connectivity will finally lead to a consensus network efficiency alteration. Moreover, the network exhibited more randomness and ipsilateral hub reorganization in patients with epilepsy [31], suggesting more synchronization and less precision of aberrant networks. It can be deduced that a less efficient network will cause larger and random activation, subsequently inducing hyperexcitability and vulnerability to external stimulation, which reflects an easy collapse during intraoperative stimulation [32,33].

Epilepsy also causes regional network disruption [32]. Further nodal analysis revealed a significant decrease in nodal efficiency and degree centrality of A6mL and A6mR in the Ep group, together with other surrounding nodes. Previous findings of structural connectivity of the supplementary motor area have demonstrated the preservation of connectivity in patients with frontal lobe epilepsy, providing evidence of the strength and stability within connections in the SMA [23]. All participants showed relatively higher degree centrality at A6mL and A6mR than at other nodes (Figure 7), indicating their importance as “hub nodes” in the motor-sensory network. Moreover, variable differences in hub nodes have been investigated in several epilepsy-related findings, demonstrating the centralization or decentralization of some nodes that play an important role in the etiology of epilepsy [34,35]. In our study, ISE patients showed a decrease in the degree centrality value in the bilateral SMA. This can be attributed to the fact that epilepsy location usually originates from the isolated cortex around the tumor mass due to subcortical impairment [36,37,38]. Therefore, the decreased degree centrality might be caused by the reduced connectivity of the isolated cortex [35]. It is supposed that the impaired connectivity with other areas and the diminished degree centrality will again induce isolation at the nodal level [36,39]. In this retrospective study, the decrease in nodal efficiency may also be due to the impaired inter-regional connection and can be attributed to the isolation of nodes. Moreover, the connection between A6mL and A6mR, as an interhemispheric connection, also showed lower FA in ISE patients, reaffirming their isolation. Consequently, it is the preserving connectivity inside nodes and external isolation that disables hub nodes from interacting with the whole network, finally leading to the segregation and distribution of network hubs. Therefore, caution should be exercised when the nodes A6mL and A6mR are involved during intraoperative stimulation because of their critical role in ISE.

## 5. Limitations

While our study clarified topological characteristics associated with the occurrence of ISE at the network level, there are still some limitations. One of the major concerns is the relatively small sample size due to the low incidence of ISE amongst the small number of patients requiring awake craniotomy. Furthermore, to maintain the consistency of the tumor location, only frontal lobe glioma was included in this study, while the network change of glioma originated from other location of the brain remains unclear. Another concern is that the limited computational power. The deterministic fiber tracking method used in this study is unlikely to address cross-fibers, which could potentially overlook microstructual white matter alternation. Finally, the single-sequence graph theory study in our study can miss subtle functional or structural alternations of brain. Thus, future studies on radiological features of ISE could combine graph theory and voxel-based analysis to reveal subtle and detailed brain dysfunction, as well as adopting more MRI sequences and advanced analytic models. Tumors of other types or same type in other locations could also be studied with the enrollment of a sufficient number of patients.

## 6. Conclusions

The SMA (A6mL and A6mR), which are likely to be the hubs of the motor-sensory network, are disconnected in ISE patients. The subsequent lower efficiency of global and regional networks might be due to hub node isolation. This finding provides a basis for presurgical radiological assessment, for which caution should be taken when it involves the nodes A6mL and A6mR during electrical stimulation.

## Figures and Tables

**Table 1:** Clinical characteristics of patients.

	Ep	nEp	Con	*p*-Value
Final sample size (n)	10	10	10	-
Age range (mean ± SE)	40.50 ± 4.50	43.80 ± 3.74	40.20 ± 2.41	0.580 *
Sex (female/male)	6/4	5/5	5/5	0.711 ^^^
Education level (yrs)	14.71 ± 1.02	15.20 ± 0.91	16.44 ± 0.83	0.232 *
Language deficits (Y/N)	3/7	3/7	/	>0.999 ^^^
Motor deficits (Y/N)	3/7	3/7	/	>0.999 ^^^
Diagnosed time (day)	60.10 ± 22.60	65.10 ± 20.96	/	0.873 ^$^
Preoperational KPS	94.00 ± 2.21	93.00 ± 2.13	/	0.749 ^$^
Histology (HGG/LGG)	4/6	5/5	/	>0.999 ^^^
IDH status (MU/WT)	4/6	3/7	/	>0.999 ^^^
Tumor volume (mL)	30.17 ± 5.15	31.25 ± 4.81	/	0.880 ^$^
Stimulation current (mA)	3.40 ± 0.49	2.85 ± 0.50	/	0.444 ^$^

Abbreviations: HGG = high-grade glioma; LGG = low-grade glioma; Mu = mutated; WT = wild type; *: one-way ANOVA; ^: fisher’s exact test; ^$^: unpaired t-test.

**Table 2:** Connections with significant inter-group differences.

Connections	Weighting	Post-Hoc *p* Value		
		Ep vs. Con	Ep vs. nEp	nEp vs. Con
A123truL and A4ulL	FA	0.017	0.071	0.089
	FN	0.001	0.781	0.002
A123truR and A4tR	FN	0.001	0.619	0.002
	FL	0.002	0.927	0.001
A6mL and A6mR	FA	0.001	0.003	0.432

A123truL: Brodmann area 1,2,3, left body trunk; A123truR: Brodmann area 1,2,3, right body trunk; A4uL: Brodmann area 4, left upper limb; A4tR: Brodmann area 4, right body trunk; A6mL: left medial area 6; A6mR: right medial area 6.

**Table 3:** Global properties with significant inter-group differences.

Global Properties	Weighting	*p* (ANOVA)	Post-Hoc *p* Value		
			Ep vs. Con	nEp vs. Con	Ep vs. nEp
Global efficiency	Binary	0.001	<0.001	0.015	0.105
	FA	<0.001	<0.001	0.004	0.031
	FN	<0.001	<0.001	<0.001	0.530
	FL	0.002	0.001	0.007	0.166
Shortest path length	Binary	0.003	0.003	0.020	0.110
	FA	<0.001	0.001	0.006	0.036
	FN	0.006	0.007	<0.001	0.245
	FL	0.003	0.003	0.016	0.073

**Table 4:** Ten nodes that exhibited nodal efficiency difference.

Node	ANOVA *p* Value (FDR Corrected)			
	Binary (0.00083)	FA Weighted (0.014)	FN Weighted (0.002)	FL Weighted (0.002)
A6m_L	0.00026	<0.001	0.002	0.002
A6m_R	0.00078	<0.001	0.002	0.002
A4t_R	0.013	0.002	<0.001	<0.001
A4ul_L	0.153	0.010	0.001	0.026
A4ul_R	0.019	0.011	<0.001	0.017
A4ll_L	0.00083	0.002	0.271	0.028
A123tru_L	0.233	0.014	0.001	0.004
A123tru_R	0.185	0.068	<0.001	0.005
A123ulhf_L	0.126	0.006	0.384	0.168
A4hf_L	0.126	0.003	0.203	0.502

**Table 5:** Seven nodes that exhibited degree centrality difference.

Node	ANOVA *p* Value (FDR Corrected)			
	Binary (0.0037)	FA Weighted (0.013)	FN Weighted (0.002)	FL Weighted (0.006)
A6m_L	<0.001	<0.001	0.002	0.002
A6m_R	<0.001	<0.001	0.001	0.002
A4t_R	0.085	0.005	<0.001	0.002
A4ul_L	0.381	0.027	<0.001	0.027
A4ul_R	0.085	0.010	0.001	0.003
A123tru_L	0.381	0.013	0.002	0.004
A123tru_R	0.213	0.027	0.001	0.006

**Table 6:** Nodal efficiency difference of A6mL and A6mR.

Node	Weighting	Post-Hoc *p* Value		
		Ep vs. Con	nEp vs. Con	Ep vs. nEp
A6m_L	Binary	0.003	0.556	0.004
	FA	0.002	0.401	0.004
	FN	0.004	0.011	0.241
	FL	0.004	0.402	0.002
A6m_R	Binary	0.002	0.556	0.007
	FA	0.001	0.264	0.005
	FN	0.004	0.009	0.332
	FL	0.003	0.211	0.023

**Table 7:** Degree centrality difference of A6mL and A6mR.

Node	Weighting	Post-Hoc *p* Value		
		Ep vs. Con	nEp vs. Con	Ep vs. nEp
A6m_L	Binary	0.004	>0.999	0.004
	FA	0.003	0.828	0.004
	FN	0.004	0.010	0.24
	FL	0.004	0.906	0.001
A6m_R	Binary	0.006	0.556	0.017
	FA	0.001	0.245	0.007
	FN	0.004	0.006	0.413
	FL	0.003	0.069	0.034

## Data Availability

Anonymized data and material will be available on reasonable request.

## Supplementary Materials

The following are available online at https://www.mdpi.com/article/10.3390/brainsci12010060/s1, Supplementary materials: Topological properties. References [19,40] are cited in the supplementary materials.
