# Supplementary material for: Topological Characteristics Associated with Intraoperative Stimulation Related Epilepsy of Glioma Patients: A DTI Network Study
Source: Brain Sci. 2021 Dec 31;12(1):60. doi: 10.3390/brainsci12010060 (PMC8774024; doi:10.3390/brainsci12010060)
Supplement: Supplementary file 1 [file brainsci-12-00060-s001.zip › brainsci-1503006-supplementary.pdf]

Supplementary materials: Topological properties.

1. Global Efficiency

Global efficiency means the ability of the information conduction of the entire topological network [19]:

$$E_{\text{glob}} = \frac{1}{N(N-1)} \sum_{i,j \in V.i \neq j} \frac{1}{l_{ij}}$$

E*glob* = global efficiency of whole functional network; *N* = the number of actual edges connecting node *i* to other nodes in the whole network; l_{ij}, *k* = the shortest path length between the node *j* and *k*.

2. Global Shortest Path Length

The shortest path length represents minimum number of passing edges during information transformation. Usually, networks with longer path length are less efficient. The calculation formula is as follows:

$$L = \frac{1}{N(N-1)} \sum_{i,j \in V.i \neq j} l_{ij}$$

where *L* = shortest path length of whole network; *N* = the number of actual edges connecting node *i* to other nodes; l_{ij} = the shortest path length between node *i* and *j*.

3. Clustering Coefficient

Cluster coefficient is an index to measure the clustering tendency of the network. Cluster coefficient represents the possibility that the neighbors of node *i* can interact with other nodes. The calculation formula as follows:

$$Ci = \frac{2ei}{ki(ki-1)}$$

where *Ci* = cluster coefficient of node *i*; *ei* = the number of actual edges connecting to other nodes; *ki* = the number of probable edges connecting to other nodes.

4. Nodal Efficiency

Nodal efficiency repersents the ability of the information conduction of a single node. The calculation formula is as follows:

$$E_{\text{nodal}} = \frac{1}{(N-1)} \sum_{i,j \in V.i \neq j} \frac{1}{l_{ij}}$$

where E_{nodal} = nodal efficiency of node *i*; *N* = the number of actual edges connecting node *i* to other nodes; l_i, *j* = the shortest path length between random nodes *i* and *j*.

5. Degree Centrality

Degree centrality(Dc) is a direct measure of single node's centrality among the entire network. Briefly, with greater degree of a node, the degree centrality of it will be relatively higher.

$$Dc = \frac{Di(g)}{N-1}$$

where Di(g) = the degree of node *i*. The degree of a nodes means the total number of nodes that connects to it. N = Total number of nodes in the entire network.

6. Small-Worldness Properties

Small-worldness properties, including gamma, lambda, and sigma, representing the efficiency of global information delivery; gamma (γ) = $C_{real}/C_{random} >> 1$ (C represented cluster coefficient), lambda (λ) = $L_{real}/L_{random} \sim 1$ (L represented shortest path length), and sigma (σ) = $\gamma/\lambda > 1$ [40]. Briefly, higher value of sigma represents higher efficiency of information delivery.
